# Peer popularity and self‐discipline as protective factors against depressive symptoms in Chinese adolescents: Do boys and girls benefit equally?

**DOI:** 10.1002/pchj.708

**Published:** 2023-12-17

**Authors:** Nan Zhu, Hui Jing Lu, Lei Chang

**Affiliations:** ^1^ Department of Psychology University of Macau Macau China; ^2^ Department of Applied Social Sciences The Hong Kong Polytechnic University Hung Hom Hong Kong

**Keywords:** depressive symptoms, gender difference, multilevel modeling, peer relationship, sex role

## Abstract

The current study examined the concurrent and longitudinal protective effects of peer popularity and self‐discipline (control, planning, and the ability to prioritize important things) against depressive symptoms among adolescents. We used multilevel modeling to examine the data of 1676 adolescents aged 12–15 years from the China Family Panel Studies (CFPS) survey, a large‐scale panel survey with a nationally representative sample. Results showed that both peer popularity and self‐discipline predicted lower levels of depressive symptoms measured concurrently. The buffering effect of self‐discipline against concurrent depressive symptoms was stronger for girls than for boys, especially in middle adolescence. Peer popularity additionally predicted lower levels of depressive symptoms 4 years later, and this effect was stronger for girls than for boys. These patterns of results were maintained after controlling for self‐rated physical health and society‐level factors. We discuss these findings against the background of distinct traditional gender roles.

According to the World Health Organization, depression constitutes one of the leading causes of illness and disability among people aged 10–19 years (World Health Organization, 2020). Clinically diagnosed depressive disorders and subclinical depressive symptoms constitute a continuum of severity, with even moderate depressive symptoms leading to impaired psychosocial functioning (Lewinsohn et al., 2000; Vijayakumar et al., 2016). Depressive symptoms in adolescence are associated with a range of adverse outcomes later in life, including poorer general health, social and work impairment, smoking, substance use, and suicidal tendencies (Keenan‐Miller et al., 2007; Maughan et al., 2013; Thapar et al., 2012).

Incidences of depression, notably in girls, rise sharply after puberty (Thapar et al., 2012). The higher susceptibility of girls compared with boys to depression typically emerges around age 13, peaks around age 15, and persists throughout adolescence (Cyranowski et al., 2000; Hyde et al., 2008; Salk et al., 2017). This gender effect is observed across cultures for both clinical diagnoses of depression and self‐reports of subclinical symptoms among healthy populations (Breslau et al., 2017; Paxton et al., 2007; Salk et al., 2017). Moreover, girls appear to be more susceptible to stress and cognitive biases related to depressive symptoms (Hankin & Abramson, 2001) but also benefit more than boys from a range of protective factors, including family connectedness, attachment, rewards for prosociality, productive coping, optimism training, and self‐acceptance and enhancement (Breton et al., 2015).

To explain the aforementioned phenomena, the current research focuses on two protective factors that have been highlighted by theories of adolescent depression in disparate literatures. Specifically, we simultaneously considered both the social protective effect derived from adolescents' popularity and status among peers and the cognitive protective effect stemming from self‐discipline (i.e., using effortful control and future‐oriented planning to suppress prepotent responses in the service of a higher goal; Duckworth & Seligman, 2006). We also took into account the potential interactions between gender and age as well as the potential roles of sociocultural factors, which allow us to integrate all these findings from the perspective of age‐specific societal pressure and gender‐role internalization.

## SOCIAL RANK THEORY AND PEER POPULARITY AS PROTECTIVE FACTOR

The social rank theory takes an evolutionary perspective on depressive symptomology, arguing that depression can be viewed as a defensive (submissive) response to disadvantaged positions and powerlessness in social rank competition, which serves to avoid harm from high‐ranking and powerful others (Gilbert, 2016; Price & Sloman, 1987; Price et al., 1994). From this perspective, concerns about losing others' favor and approval, or lacking valued abilities (e.g., physical attractiveness, academic skills) give rise to the perception of submissive status, causing one to feel inferior and insecure (Gilbert, 2016). Gaining social approval and popularity offers considerable advantages throughout human evolution. However, sometimes when individuals are trapped in socially unrewarding situations or face powerful foes in social rank competition, it is adaptive to concede defeat to limit social punishment. Signs of competitive disadvantage might trigger unconscious, involuntary affective and behavioral responses (e.g., behavioral withdrawal, low self‐esteem, anhedonia) that characterize depression, which is seen as such adaptive behavioral display (Price et al., 1994). For example, low‐ranking persons, compared with their higher‐ranking peers, might be more sensitive to others' opinions (whether he/she is being looked down upon), more likely to have low self‐esteem, and more likely to display submissive defensive behaviors (e.g., Gilbert, 2000, 2016; Irons & Gilbert, 2005). Conversely, higher‐ranking adolescents are less likely to find themselves in submissive and depression‐inducing situations. Indeed, past research has shown that peer popularity and status buffer against depressive symptomology, whereas peer rejection increases the risks of depression (Irons & Gilbert, 2005; Kiesner, 2002; La Greca & Harrison, 2005; Oldehinkel et al., 2007). Depressive symptoms were associated with social anxiety, shame, submissive behaviors, and unfavorable social comparisons (Allan et al., 1994; Gilbert, 2000). Peer status was found to be a longitudinal predictor of reduced depressive symptoms (Kiesner, 2002), whereas peer rejection generally predicts greater depressive symptomatology, although specific types of rejection were linked to specific symptoms (e.g., aggressive‐rejected youths were higher in feelings of ineffectiveness, whereas neglected and submissive‐rejected youths exhibited higher anhedonia; Hecht et al., 1998). Research also indicates that low‐popularity adolescents with social‐rank concerns are especially at risk of depression (Irons & Gilbert, 2005). By contrast, high peer status and peer popularity exhibit protective effects against depressive symptoms among at‐risk individuals (e.g., racial minority students affected by microaggressions; Lilly et al., 2018).

Importantly, the social rank theory offers one compelling explanation of adolescent gender differences in depression susceptibility. During the pubertal transition, boys and girls both experience heightened affiliative needs and sensitivity to interpersonal and social relationships (Cyranowski et al., 2000). From the perspective of social rank theory, this is because puberty marks the onset of mating competition in human evolutionary history, which is especially intense for young males (Cummins, 2005). This might drive up the sensitivity of boys to peer popularity. Indeed, adolescent boys with more friends are less vulnerable to interpersonal, depression‐inducing stressors in their peer groups (Fournier, 2009; Irons & Gilbert, 2005; Price et al., 1994). Girls, by contrast, do not seem to benefit from peer acceptance and support as readily as boys do (Breton et al., 2015). Nevertheless, one longitudinal study found that childhood peer status predicted women's risk of depression 30 years later (Modin et al., 2011), implying that peer popularity is by no means unimportant for girls. It actually might have long‐lasting effects by boosting long‐term positive self‐regard.

Rather than from peer group ranks, adolescent girls seem to benefit more from social support in terms of the number of people they talk to when feeling stressed (Schraedley et al., 1999). Engagement in rewarding social activities appears to reduce susceptibility to depressive symptoms to a greater degree in girls than in boys (Ryba & Hopko, 2012). Eberhart et al. (2006) found that more positive self‐perception in interpersonal domains acted as a protective factor against depressive symptoms in girls. Therefore, peer popularity might also provide protective effects against depression for girls, albeit in different ways (e.g., reinforcing positive self‐regard). By weighing the existing evidence, we hypothesized that peer popularity should be associated with lower severity of depressive symptoms in both girls and boys.

## COGNITIVE THEORIES AND SELF‐DISCIPLINE AS PROTECTIVE FACTOR

Unlike the social rank theory, which focuses on social dynamics, cognitive theories of depression (LeMoult & Gotlib, 2019) emphasize negative cognitive biases (and the lack of positive biases) about the self, the world, and the future as the central characteristics of depression and risk for depression (Beck, 2002). It is believed that deficits in cognitive control lead to the inability to dispel such negative thoughts and biases, which constitutes cognitive vulnerability that, in the presence of stressors, produces depressive symptoms (Abramson et al., 1989; Hyde et al., 2008). In support of this, deficits in cognitive control and coping abilities have been linked to increased depression risk and persistence of depressive episodes (Joormann & Vanderlind, 2014).

Many cognitive traits, such as self‐esteem, hope, and problem‐solving capacity, are associated with lower severity of depressive symptoms or lower chances of major depressive disorder (Cong et al., 2021; Luo et al., 2016; Orth et al., 2014; Zhou et al., 2020). All these factors are related to self‐discipline, which is captured by a range of cognitive characteristics, such as attentiveness, low impulsivity, task persistence, and future‐oriented planning (Duckworth & Seligman, 2005). We argue that self‐discipline might function as a protective factor in multiple ways. First, self‐discipline is underlain by cognitive abilities to suppress predominant responses (Duckworth & Seligman, 2006), which indicates efficient cognitive control functioning. This should allow individuals to steer away more easily from negative thoughts and devote cognitive resources to adaptive coping strategies. Second, individuals with greater self‐discipline are less likely to give up or adopt an avoidant strategy when faced with everyday problems (which can turn into depression‐inducing stressors) and are more likely to tackle the problems with persistent efforts and planning. Hence, self‐discipline might protect healthy individuals from depressive symptoms by directly removing the source of stressors through efficient problem‐solving (Sobol et al., 2021). This contrasts with cognitive coping strategies (e.g., reappraisal), which serves as a compensatory mechanism that attenuates depressive symptoms for individuals who use maladaptive strategies to deal with stress, but not for other healthy individuals (Joormann & Vanderlind, 2014). Most other cognitive protective factors also do not contribute to the removal of the source of depression.

Importantly, self‐discipline seems to benefit girls more than boys during middle adolescence. Research has demonstrated that females, compared with males, are more susceptible to negative attributional styles and tend to engage in increased rumination about interpersonal events and body image, especially during adolescence (Morken et al., 2019; Rawana & Morgan, 2014). Such vulnerabilities can be countered by greater self‐discipline. In the meantime, Duckworth and Seligman (2006) showed that the phenomenon of girls typically outperforming boys academically is mainly due to cognitive differences (better‐developed self‐discipline) rather than IQ. In other words, girls tend to concentrate their cognitive abilities on academic tasks rather than getting distracted like boys often do, and such self‐discipline can also be used to deal with depression‐inducing stressors. Indeed, research has shown that higher cognitive ability (which is essential for self‐discipline) buffered the relation between stress and greater depressive symptoms for girls but not for boys (Riglin et al., 2016). Additionally, personal attitudes that are conducive to self‐discipline, such as focusing on the positive and future optimism, also had greater protective effects for girls than for boys (Breton et al., 2015). Breton et al. (2015) additionally found that productive coping strategies, such as problem‐solving and focusing on achievements, were associated with lower severity of depressive symptoms among girls but not among boys in a nonclinical community population. From this perspective, self‐discipline might be a stronger cognitive protective factor for girls than for boys.

## INTEGRATING DIFFERENT PERSPECTIVES ON GENDER DIFFERENCES IN DEPRESSION SUSCEPTIBILITY

The previous analysis showed that both peer popularity and self‐discipline are linked to established theoretical frameworks that explain the risks, etiology, and symptomology of depression. Although generally viewed as separate mechanisms in early research, social and cognitive protective factors of depression have been integrated into some research (no such research has simultaneously examined peer popularity and self‐discipline, however). For example, studies have shown that a perceived sense of control, a cognitive factor that underlies self‐discipline, mediates the effects of social status and education on depressive symptoms (Lachman & Weaver, 1998; Mirowsky & Ross, 1990; Ross & Mirowsky, 2006). Cognitive coping strategies, such as positive reappraisal and planning, were also found to mediate the protective effects of social support against depressive symptoms in healthy adolescents (Sobol et al., 2021).

Certain features of the societal context facing Chinese adolescents also prompt us to include both peer popularity and self‐discipline in our model of protective factors against depression. To begin, self‐discipline is closely linked to academic excellence in a highly competitive environment (Duckworth & Seligman, 2006). As academic achievement and education are highly valued in the competitive Chinese society (Zhu & Chang, 2019a), adolescents with greater self‐discipline and better academic performance are also more likely to be admired by peers. In the meantime, peer rejection and low peer status might impair adolescents' emotional regulation and induce social anxiety (Gilbert, 2000; Joormann & Vanderlind, 2014), which deplete cognitive resources that can otherwise contribute to self‐discipline. This partly intertwined relationship might lead one to overestimate one protective effect without controlling for the other. Thus, the current research aims at generating empirical evidence that would allow us to integrate previously independent theoretical frameworks (the social rank and cognitive theories). Moreover, researchers have increasingly recognized that the gender difference in the susceptibility to depressive symptoms is caused by a convergence of genetic proclivities, developmental changes in affective responding, cognitive abilities, social adjustment, and sociocultural challenges. All these factors (rather than a single mechanism) might manifest differentially in the lives of boys and girls during early to middle adolescence, contributing to age‐specific patterns of gender differences in depression incidences and depressive symptoms (Cyranowski et al., 2000; Hyde & Mezulis, 2020). In support of this view, Salk et al. (2017) conducted meta‐analyses on data sets from more than 90 countries and concluded that gender differences in clinical diagnoses and self‐rated symptoms of depression both peaked in middle adolescence (odds ratio = 3.02 for ages 13–15 years, and overall effect size *d* = 0.47 for age 16 years). Thus, the transition period from early to middle adolescence is crucial. In the present study, we investigated potential gender differences in depressive symptoms among adolescents who were 12–15 years old when their levels of depressive symptoms were first measured. This age range covers the well‐documented changes in the gender difference in depressive symptoms (Salk et al., 2017). We investigated the interactions between age and protective factors and between age and gender. Specifically, from a sociocultural perspective, the social pressures generated by stereotypical gender roles tend to increase with age for both genders during adolescence (Bone et al., 2020; Larson & Richards, 1989). In particular, the higher risk of depressive symptoms in girls might be mediated by body dissatisfaction (Morken et al., 2019), which constitutes a gender stereotype that poses a greater threat to the self‐esteem of girls than to that of boys. Based on extant findings regarding the age pattern in depressive symptoms between the two genders and the aforementioned sociocultural analysis, we hypothesized that gender differences in depressive symptoms would be more prominent in middle than early adolescence.

Although gender differences in depressive symptoms in adolescents are observed across most cultures and ethnicities, the effect sizes may vary considerably across societies and regions (Hilt & Nolen‐Hoeksema, 2014) due to factors such as socioeconomic development, education, and gender inequity (Salk et al., 2017). Male‐skewed sex ratios are regarded as one of the key indicators of gender inequity (Jayachandran, 2015), which has been suggested to increase the susceptibility of female adolescents to depressive symptoms (Bone et al., 2020; Hilt & Nolen‐Hoeksema, 2014). However, sex ratio as a proxy for gender inequity might affect cultures differently. Zhou et al. (2013), for example, attributed the increased depressive symptoms among Chinese men to the highly male‐skewed sex ratio, as this purportedly intensified intrasexual competition among men. Another social context to be considered is educational attainment, which constitutes a protective factor against depression (Colman et al., 2014) but is also a major source of stress for children and adolescents due to fierce academic competition (Quach et al., 2015), which is particularly relevant in a Chinese cultural context (Zhu & Chang, 2019a). Past research has shown that a stronger parental educational background is correlated with a lower risk of depression (Wang et al., 2016). Furthermore, average years of education are associated with modern, egalitarian values, such as gender equality (Newson & Richerson, 2009). Based on previous research and theorization, the current study included local sex ratio and average years of education as society‐level predictors. In general, we expected that a male‐skewed sex ratio would be associated with a higher prevalence of depressive symptoms, whereas average years of education would predict a lower prevalence of depressive symptoms, especially among female adolescents.

To summarize, the aforementioned reasoning leads to the following hypotheses:

Hypothesis 1 Both peer popularity and self‐discipline should be associated with lower severity of depressive symptoms.

Hypothesis 2 The protective effect of peer popularity on depressive symptoms may differ between female and male adolescents.

Hypothesis 3 The protective effect of self‐discipline on depressive symptoms should be stronger for female adolescents than for male adolescents.

Hypothesis 4 A gender difference in depressive symptoms is more likely to manifest among older adolescents than among younger adolescents.

The current research addressed these issues by using the China Family Panel Studies (CFPS; Xie, 2012) dataset, which sampled both adults and children in the majority of Chinese provinces. The CFPS data set used the Center for Epidemiologic Studies Depression Scale (CES‐D; Radloff, 1977) to indicate the severity of depressive symptoms among adolescents as a continuous variable. This is consistent with our purpose to examine depressive symptoms, rather than clinically diagnosed major depression disorder. The present study examined both concurrent and longitudinal effects of the same set of predictors on depressive symptoms but did not make different predictions regarding the longitudinal effects.

## METHOD

### Data

The current study used data from the CFPS, a large‐scale, longitudinal panel survey project conducted by the Institute of Social Science Survey at Peking University (extensive information about the survey can be found at www.isss.edu.cn/cfps/). CFPS surveys collected representative samples from 25 provinces that represent 95% of the population in mainland China (Xie, 2012). Beginning in 2010, the CFPS team conducted a full survey every 2  years, with 14,798 households surveyed in the first wave. The CFPS sought to understand social, economic, educational, and psychological changes by collecting a vast amount of data from areas ranging from family income and social relationships to individuals' physical and mental health (publicly available information and data of the CFPS can be found at www.isss.pku.edu.cn/cfps). For each wave, different individual‐level questionnaires were administered to different age groups. Participants aged 10–15 years responded to the child questionnaire by themselves. Participants aged 16 years or above responded to the adult questionnaire.

The current study used data from participants who completed the child questionnaire in 2012 (CES‐D was not measured in the baseline survey of 2010). We additionally collected the responses of these participants to the CES‐D scale in 2016, which was part of the adult questionnaire in 2016. After excluding participants with missing responses, the final samples comprised 1676 participants at Time 1 (2012), including 835 females and 841 males who were 12 to 15 years old in 2012 (*M*_age_ = 13.49 years, *SD* = 1.13), and 1243 participants at Time 2 (2016), including 611 males and 632 females (*M*_age_ = 17.67 years, *SD* = 1.22). The CFPS data did not specify the ethnicity of the participants, because the vast majority of the population in the areas being sampled were ethnic Chinese (including both Han Chinese and minorities). The gender ratio (49.8% females at Time 1 and 50.8% females at Time 2) and provincial distribution of the current subsample after excluding missing responses were very similar to those of the original sample (the entire child sample in 2012, *N* = 8620) it is drawn from (47.5% females; see Table S1 for more details). Overall, the current subsample closely resembled the composition and regional representativeness of the original sample, even though our sample was limited to a certain age group (adolescents aged 12 to 15 years in 2012). Further, independent samples *t*‐tests revealed that adolescents who were excluded because of missing responses on one or more scales (*N* = 185) did not differ from the current subsample in terms of age (*p* = .437) or depressive symptoms (*p* = .382).

The multilevel data structure of the CFPS allowed us to delineate the effects of individual differences and social contexts. To maximize its geographical representation, the CFPS used a three‐stage probability sample based on implicit stratification (the detailed sampling design is reported in Xie & Hu, 2014). Specifically, CFPS chose to focus on five typical provinces (Shanghai, Liaoning, Henan, Gansu, and Guangdong) located in different regions of China. These five typical provinces constituted five sampling frames, whereas 20 other provinces were combined into a sixth sampling frame. In the subsequent sampling stages, the CFPS used counties, which are administrative units directly below provinces in China, as the primary sampling unit (except in Shanghai, where a lower‐level administrative unit was used; we refer to all these primary sampling units as counties). The CFPS also collected county‐level data on demographics and socioeconomic development (including local sex ratio and average years of education) in 2010 for all 176 counties being sampled. In the current study, participants were nested within 157 and 153 county‐level clusters for Model 1 and Model 2, respectively (cluster sizes ranged from 1 to 42, *M* = 12, *SD* = 8.25).

### Ethical approval

Since this study used only openly accessible archival data, an ethical approval was not required.

### Measures

All measures are presented in the native language of the participants (Simplified Chinese). As the CFPS study was not originally designed for the purpose of examining the protective effects of peer popularity and self‐discipline, researchers first combed through the child questionnaire in 2012 in search of relevant items. Only highly relevant items were used to form the indices and variables used in our final analysis. Although it is beyond the scope of this study to conduct comprehensive validations of the measures that we chose, we examined the correlations between these measures and other candidate items and indices. The results, reported in detail in Data S1, largely attest to the construct validity of the measures used here.

#### Dependent variables: Depressive symptoms (Time 1 and Time 2)

Depressive symptoms were measured at both Time 1 (2012) and Time 2 (2016) using the CES‐D (Radloff, 1977), which has been validated and widely used in the Chinese cultural context (e.g., Zhu et al., 2021). The scale consisted of 20 self‐report items asking participants to estimate the frequency of depression‐like symptoms (e.g., “I felt depressed,” “I felt that everything I did was an effort”). These items were rated on a 4‐point scale (1 = *rarely or less than 1 day*, 2 = *some of the time or 1–2 days*, 3 = *a moderate amount of the time or 3–4 days*, 4 = *most or all of the time or 5–7 days*). Average ratings of the 20 CES‐D items were used to represent the depressive symptoms of the participant instead of the total scores because this allows us to maximize the use of the data (participants with one or two missing responses were not excluded, although their total score was missing). However, the results were extremely similar regardless of which dependent measure we used. The alpha coefficients for this scale were .81 and .82 at Time 1 and Time 2, respectively.

#### Independent variable: Peer popularity (Time 1)

Peer popularity was measured using two self‐report items, which were rated on an 11‐point scale ranging from 0 to 10: “How popular are you?”, “How well are you getting along with others?”. The average score of the two items, which were strongly and positively correlated (*r* = .58), was used to indicate peer popularity, with a higher average score representing a higher self‐perception of peer popularity.

#### Independent variable: Self‐discipline (Time 1)

Self‐discipline (e.g., “I do things carefully,” “I finish homework before playing”) was measured using a 12‐item “self‐discipline scale” (Xie, 2012), which was conceptually similar to established psychological scales measuring the construct of “insight, planning, and control” (e.g., Figueredo, 2007) and self‐discipline (Duckworth & Seligman, 2005). These items were rated on a 4‐point scale ranging from 1 (*strongly disagree*) to 4 (*strongly agree*). The average score was used to indicate the degree to which the participants rely on self‐confidence and planning to overcome obstacles and achieve desirable outcomes. The alpha coefficient for this measure was .80.

#### Individual‐level controls: Health (Time 1)

Physical health was suggested by previous studies to strongly predict depressive symptoms in Chinese samples (e.g., Wang et al., 2016). Therefore, a single self‐report item (“How do you think your physical health is?”) rated on a 5‐point scale (1 = *poor health*, 5 = *very good health*), was included as a control variable.

#### County‐level controls: Sex ratio and average years of education

We also considered potential social contexts that might influence the protective factors on adolescent depressive symptoms by controlling for certain statistics at the county level (which was the second and main sampling level of the CFPS data set). Specifically, we included the population sex ratio between ages 10 and 19 years (number of males per 100 females) and average years of education as county‐level control variables. These county‐level statistics were collected by the CFPS team in 2010.

### Statistical analyses

The CFPS data conforms to a multilevel structure with data of each individual defined as Level 1. All individuals were nested within different counties, defined as Level 2. Therefore, multilevel analyses were conducted using the HLM 8 software (Raudenbush et al., 2019), which allowed us to consider regional variations that might lead to varying patterns of depressive symptoms across different counties. We first examined the baseline models that estimate the variances of level‐1 residuals (σ^2^) and level‐2 (county‐level) residuals (τ_00_) without the predictors. For both Time 1 (2012) and Time 2 (2016), the Level 2 variance components were significant for depressive symptoms (Time 1: τ_00_ = 0.0126, χ^2^(156) = 401.14, *p* < .001; Time 2: τ_00_ = 0.0061, χ^2^(153) = 227.10, *p* < .001). The intra‐class correlation coefficients (ICCs) were .12 for depressive symptoms at Time 1 and .05 for depressive symptoms at Time 2, meaning that cross‐county variability accounted for 12% and 5% of the total variability in depressive symptoms at Time 1 and Time 2, respectively. ICC values between .05 and .20 are considered common in social research studies using multilevel models (Peugh, 2010).

We then tested two multilevel models (Model 1 and Model 2), which were identical in predictors but differed in the dependent measure: Model 1 used depressive symptoms in 2012 (when participants were 12 to 15 years old and responded to the child questionnaire) whereas Model 2 used depressive symptoms in 2016 (when participants were 16 to 20 years old and responded to the adult questionnaire). Thus, Model 2 allowed us to investigate the longitudinal effects of the predictors on depressive symptoms in a slightly smaller sample. In both models, depressive symptoms were regressed on level‐1 (individual‐level) predictors, including gender (binary‐coded: 0 = *female*, 1 = *male*), age in 2012 (as a continuous variable), self‐rated health, social and cognitive protective factors (i.e., peer popularity and self‐discipline), as well as the interactions between gender and the two protective factors, between age and protective factors, and between gender and age. All level‐1 predictors were estimated as fixed effects only (allowing free estimation of random effects may result in the models failing to converge). Among them, continuous variables were all group‐mean‐centered. To quantify how well these individual‐level predictors accounted for the variance of depressive symptoms within counties, we calculated the proportional reduction in the variance of level‐1 residual σ^2^ (Peugh, 2010).

Proportional reduction in σ^2^ = $\left({\sigma^{2}}_{\text{Baseline}}-{\sigma^{2}}_{\text{Level}\;1}\right)/{\sigma^{2}}_{\text{Baseline}.}$

For both models, we further regressed the overall intercepts and the slopes of gender, respectively, on grand‐mean‐centered level‐2 (county‐level) predictors, namely sex ratio and average years of education. To quantify the amount of variance in adolescent depressive symptoms accounted for by county‐level predictors, we calculated the proportional reduction in the variance of level‐2 residual τ_00_ (Peugh, 2010).

Proportional reduction in τ_00_ = $\left({\tau_{00}}_{\text{Baseline}}-{\tau_{00}}_{\text{Full Model}}\right)/{\tau_{00}}_{\text{Baseline}}$.

## RESULTS

Correlations among individual‐level variables and the descriptive statistics are presented in Table 1. Depressive symptoms were moderately and positively skewed at Time 1 (skewness = 1.00, 0.81, respectively, for males and females) and Time 2 (skewness = 0.82, 0.93, respectively, for males and females). Independent samples *t*‐tests revealed that females, compared with males, reported higher self‐discipline, *t*(1675) = 3.97, *p* < .001, 95% CI [0.05, 0.15]. There was no gender difference in age, peer popularity, self‐rated health condition, or depressive symptoms at either time point, *p*s > .050. At the individual level, peer popularity and self‐discipline were positively correlated with each other, both positively correlated with self‐rated health, and both negatively correlated with depressive symptoms at Time 1. Peer popularity also had a weak but significant correlation with depressive symptoms at Time 2. Self‐rated health in 2012 was negatively correlated with depressive symptoms at both time points. None of these variables have a sizable correlation with the age of participants. At the county level, sex ratio (*M* = 111.75, *SD* = 10.05, range = 82.23 to 163.22) and average years of education (*M* = 8.96, *SD* = 1.36, range = 4.52 to 13.10) were negatively correlated (*r* = −.14, *p* < .001).

**TABLE 1:** Pearson correlations among individual‐level variables and descriptive statistics.

Variables	Sex	Age	Peer popularity	Self‐discipline	Health	CES‐D 2012	CES‐D 2016
Age	−.01	‐					
Peer popularity	−.03	−.02	‐	‐			
Self‐discipline	−.10***	−.07**	.26***	‐			
Health	<.01	−.01	.24***	.09***	‐		
CES‐D 2012	−.03	−.05*	−.27***	−.18***	−.22***	‐	
CES‐D 2016	−.04	.01	−.07*	−.03	−.10**	.23***	‐
*M* (*SD*)							
Male	‐	13.49 (1.11)	7.18 (1.70)	3.49 (0.53)	3.84 (0.93)	1.56 (0.32)	1.52 (0.36)
Female	‐	13.50 (1.14)	7.29 (1.72)	3.60 (0.53)	3.83 (0.92)	1.58 (0.32)	1.55 (0.37)

*Note*: Abbreviation: CES‐D, Center for Epidemiologic Studies Depression Scale.

* *p* < .05.

** *p* < .01.

*** *p* < .001.

The results of the multilevel analysis are reported in Tables 2 and 3. For Model 1 (Table 2), level‐1 predictors accounted for 10% of the individual‐level variance in depressive symptoms in 2012, compared with baseline (from 0.0898 to 0.0805). We detected a weak but significant gender difference in depressive symptoms (*B* = −0.0298, *p* = .047) and a significant effect of age (*B* = 0.0276, *p* = .001), indicating that older adolescents were more susceptible to depressive symptoms. These effects were qualified by a significant and negative gender‐by‐age interaction (*B* = −0.0309, *p* = .023), implying a greater gender gap in depressive symptoms among older rather than younger adolescents. Health, peer popularity, and self‐discipline were associated with lower levels of depressive symptoms (*B*s = −0.0412, −0.0369, −0.1208, *p*s < .001). The interaction between gender and self‐discipline was positively associated with depressive symptoms in 2012 (*B* = 0.0586, *p* = .034), whereas the interaction between gender and peer popularity was not significant (*B* = 0.0090, *p* = .294). This means that self‐discipline had a stronger protective effect against depressive symptoms for girls than for boys. The interactions between age and peer popularity (*B* = −0.0072, *p* = .051), and between age and self‐discipline (*B* = 0.0186, *p* = .144) were not significant. Level‐2 predictors explained an additional 12% of the cross‐county variance in depressive symptoms in Model 1 (from 0.0135 to 0.0119). Average years in education negatively predicted depressive symptoms (*B* = −0.0222, *p* = .041), whereas sex ratio did not have a significant effect on depressive symptoms (*B* = 0.0026, *p* = .080). Neither of the level‐2 variables had a significant effect on the slope of gender, *p*s > .050.

**TABLE 2:** Results of Model 1: Predicting depressive symptoms in 2012.

Predictors	*β*	*SE*	95% CI		*t*‐ratio
			Lower limit	Upper limit	
Level 1 (individual level)					
Gender	−0.0298	0.0150	−0.0592	−0.0004	−1.99*
Age	0.0276	0.0086	0.0108	0.0444	3.22**
Health	−0.0412	0.0085	−0.0579	−0.0246	−4.85***
Peer popularity	−0.0369	0.0063	−0.0492	−0.0247	−5.89***
Self‐discipline	−0.1208	0.0218	−0.1635	−0.0782	−5.56***
Gender × Peer popularity	0.0090	0.0086	−0.0078	0.0257	1.05
Gender × Self‐discipline	0.0586	0.0276	0.0045	0.1128	2.12*
Age × Peer popularity	−0.0072	0.0037	−0.0145	0.0000	−1.95
Age × Self‐discipline	0.0186	0.0127	−0.0063	0.0436	1.46
Gender × Age	−0.0309	0.0136	−0.0575	−0.0043	−2.27*
Level 2 (county level)					
Effect on intercept: Sex ratio	0.0026	0.0015	−0.0003	0.0055	1.76
Effect on intercept: Average years of education	−0.0222	0.0108	−0.0433	−0.0011	−2.06*
Effect on gender slope: Sex ratio	−0.0023	0.0015	−0.0052	0.0007	−1.52
Effect on gender slope: Average years of education	−0.0076	0.0108	−0.0287	0.0136	−0.70

*Note*: All Level 1 continuous variables were group‐mean‐centered. Level 2 variables were grand‐mean‐centered.

* *p* < .05.

** *p* < .01.

*** *p* < .001.

**TABLE 3:** Results of Model 2: Predicting depressive symptoms in 2016.

Predictors	*β*	*SE*	95% CI		*t*‐ratio
			Lower limit	Upper limit	
Level 1 (individual level)					
Gender	−0.0313	0.0203	−0.0710	0.0085	−1.54
Age	−0.0086	0.0136	−0.0353	0.0181	−0.63
Health	−0.0146	0.0135	−0.0410	0.0118	−1.09
Peer popularity	−0.0208	0.0099	−0.0403	−0.0013	−2.10*
Self‐discipline	−0.0213	0.0332	−0.0865	0.0438	−0.64
Gender × Peer popularity	0.0294	0.0136	0.0027	0.0560	2.16*
Gender × Self‐discipline	−0.0196	0.0416	−0.1012	0.0620	−0.47
Age × Peer popularity	−0.0114	0.0061	−0.0235	0.0006	−1.86
Age × Self‐discipline	−0.0018	0.0197	−0.0405	0.0368	−0.09
Gender × Age	0.0184	0.0213	−0.0234	0.0602	0.86
Level 2 (county level)					
Effect on intercept: Sex ratio	−0.0001	0.0015	−0.0031	0.0029	−0.07
Effect on intercept: Average years of education	−0.0115	0.0125	−0.0359	0.0130	−0.92
Effect on gender slope: Sex ratio	−0.0010	0.0018	−0.0044	0.0025	−0.54
Effect on gender slope: Average years of education	−0.0198	0.0169	−0.0529	0.0133	−1.17

*Note*: All Level 1 continuous variables were group‐mean‐centered. Level 2 variables were grand‐mean‐centered.

* *p* < .05.

For Model 2 (Table 3), level‐1 predictors accounted for 1% of the individual‐level variance in depressive symptoms in 2016, compared with baseline (from 0.1289 to 0.1281). Only peer popularity (*B* = −0.0208, *p* = .036) and the interaction between gender and peer popularity (*B* = 0.0294, *p* = .031) were found to significantly and longitudinally predict depressive symptoms in 2016. Thus, the protective effect of peer popularity appeared to persist even after 4 years. Moreover, this protective effect was stronger among females than among males. The other level‐1 predictors were not significant, *p*s > .050. Level‐2 predictors explained an additional 18% of the county‐level variance of depressive symptoms in Model 2 (from 0.0046 to 0.0038). However, no level‐2 predictor was significant, *p*s > .050.

To further investigate the interaction between age and gender on depressive symptoms in 2012, given a potential qualitative change in the pattern of gender difference in depressive symptoms around the ages of 13 and 14 years, we divided the 2012 sample into two subsamples: the younger group was 12 or 13 years old (born in 2000 or 1999; *n* = 854, including 419 females) and the older group was 14 or 15 years old (born in 1998 or 1997; *n* = 824, including 418 females) in 2012. Separate multilevel analyses were conducted on these two groups. The models were similar to Model 1 except that age and interactions involving age were not included as a level‐1 predictor. The detailed results of the multilevel analyses for the younger and older subsamples are presented in Tables S2 and S3, respectively, in Supporting Information. As expected, we only found significant gender differences (*B* = −0.0842, *p* < .001; females reported higher depressive symptoms than males) and interaction between gender and self‐discipline (*B* = 0.0699, *p* = .034; indicating that self‐discipline had a stronger protective effect against depressive symptoms for girls than for boys) in the older group, but not the younger group (*p*s > .050). In neither subsample did we find a significant interaction between gender and peer popularity (*p*s > .050). At Level 2, we found that for the younger group (*B* = −0.0339, *p* = .007), but not the older group (*B* = −0.0162, *p* = .277), average years of education was associated with lower depressive symptoms, indicating that younger adolescents in better‐educated counties were at lower risks for depressive symptoms.

## DISCUSSION

Adolescent boys and girls face unique challenges during and after the pubertal transition, which leads to increased risks of depressive symptoms (Hyde & Mezulis, 2020). They also differentially benefit from various protective factors (Breton et al., 2015). This research is an attempt to integrate two theoretical perspectives on depressive symptoms and risks, one derived from the social rank theory (Gilbert, 2000, 2016) and the other derived from cognitive theories (Beck, 2002; LeMoult & Gotlib, 2019). Using the CFPS data, we focused on the protective effects of peer popularity and self‐discipline among Chinese girls and boys aged 12 to 15 years. We discovered that both key variables were associated with lower levels of concurrent depressive symptoms, supporting Hypothesis 1 and our reasoning based on both the social rank theory and cognitive theories of depression.

Higher peer popularity predicted lower depressive symptoms measured concurrently as well as 4 years later, supporting the social rank perspective. However, the protective effect of peer popularity on concurrent depressive symptoms did not differ between genders. Thus, Hypothesis 2 is not supported. Nevertheless, this does not necessarily challenge the social rank theory. Any gender difference in the protective effect of peer popularity, according to social rank theory, should be contingent on each gender's exposure to intense social rank competition (Fournier, 2009; Price et al., 1994). The expectation that the peer‐popularity effect should be more prominent for boys is backed by the fact that boys’ testosterone changes are correlated with their peer status, which is consistent with males' greater involvement in aggression and social rank competition in human evolutionary history (Cummins, 2005). However, when aggressiveness and dominance are socially disapproved and both genders face similar competitive pressure and source of popularity (which is largely dependent on academic performance, rather than assertiveness and leadership in peer groups, for instance) due to the heightened emphasis on academic achievement in the Chinese context, the gender difference in peer‐popularity effect should vanish. This seems to explain our finding regarding Hypothesis 2.

We also found that peer popularity was a stronger protective factor for girls than for boys against depressive symptoms 4 years later. This seems to suggest that girls, but not boys, benefitted from peer popularity as a long‐term buffer against depressive symptoms. This might be attributed to the different types of peer popularity involved for girls than for boys (e.g., stable friendships rather than group popularity). Females' greater emphasis on interpersonal relationships and intimacy might allow them to develop deeper and more meaningful peer relationships and friendships than males do (Hamilton et al., 2015), which provides buffers against future depressive stressors. Alternatively, peer popularity might build up positive self‐perception for girls (Eberhart et al., 2006), which attenuates the risks of depressive symptoms in the long run.

Consistent with Hypothesis 3, self‐discipline had a stronger negative effect on concurrent depressive symptoms for girls than for boys, although the effect size of this interaction is small. Reasons for the small effect might be that (1) there is a relatively small overall gender effect among Chinese adolescents, reducing gender differences in cognitive vulnerability (Hyde et al., 2008), which, in turn, reduced the protective effect of self‐discipline; (2) only older adolescents demonstrated this gender effect; and (3) the inclusion of many covariates allowed us to test the hypothesis in more stringent settings. Given these reasons, it would be premature to dismiss the current finding as merely an artifact generated by high statistical power produced by a relatively large sample. Still, the directionality of the result is compatible with extant findings that cognitive protective factors (e.g., self‐esteem, productive coping strategies, general cognitive abilities) reduce stress‐induced depressive symptoms and risks of major depression disorder to a greater degree for girls than for boys (e.g., Breton et al., 2015; Riglin et al., 2016). However, the protective effect of self‐discipline did not extend to depressive symptoms measured 4 years later.

The current research only found relatively small gender effects. For depressive symptoms assessed in late adolescence to young adulthood, we did not find a significant gender difference after controlling for other predictors. This is consistent with past research on Chinese adolescents, which typically showed smaller gender differences in early and middle adolescence (e.g., Tepper et al., 2008; Wang et al., 2016) compared with those in other cultures (Salk et al., 2017). In a meta‐analysis, for instance, Tang et al. (2019) reported that the pooled event rates of depressive symptoms across 51 studies conducted in China were .262 for boys and .275 for girls. Additionally, we found a small but significant gender‐by‐age interaction on depressive symptoms measured concurrently (in 2012), supporting Hypothesis 4. The expected gender effect (females were more at risk for depressive symptoms) was only present among older adolescents (14‐ and 15‐year‐olds) but not younger adolescents (12‐ and 13‐year‐olds). Our analyses of separate models for younger and older groups (reported in Supportive Information) also found that a stronger protective effect of self‐discipline for girls than for boys was present in the older group (14 and 15 years old), but not in the younger group (12 and 13 years old). These patterns of results were maintained after we controlled for self‐rated health and higher‐level social context variables. Culture‐specific social pressures faced by Chinese adolescents might contribute to a smaller gender difference in depressive symptoms as well as self‐discipline effects. Chinese education and parenting indoctrinate a focus on academic achievement for both genders equally in part because of the single‐child policy that ended only recently. Thus, girls in China may be encouraged or pressured to exert even greater self‐discipline than boys to achieve academic excellence. The academically oriented self‐discipline may also contribute to the lessening of the girls' vulnerability to depressive symptoms. Consistent with Duckworth and Seligman (2006), we found that girls reported higher self‐discipline than boys did (Table 1).

One unifying mechanism for the gender difference in the effects of the two protective factors as well as macro‐level sociocultural factors might have to do with the interaction between the degree of internalization of traditional gender roles in adolescence and peer dynamics. Social pressures to conform to stereotyped gender roles become salient during adolescence (Larson & Richards, 1989), and exposure to same‐sex peers might strengthen and exaggerate traditional gender roles that increase female vulnerability to depression (Rose & Rudolph, 2006). Specifically, girls tended to be socialized to focus on relationships, intimacy, and communion rather than personal autonomy, instrumentality, or agency (Cyranowski et al., 2000). An overemphasis on communion and lack of agency might lead to less of a sense of control, which has been linked to higher rates of depression (Lachman & Weaver, 1998; Mirowsky & Ross, 1990; Steptoe et al., 2007). Research has shown that a perceived sense of control mediates the effects of social status and education on depressive symptoms (Lachman & Weaver, 1998; Mirowsky & Ross, 1990; Ross & Mirowsky, 2006). Importantly, this might not only explain the varying gender gaps in the susceptibility to depressive symptoms across societies but also the age pattern of such gender gaps. Older adolescents are more likely to be exposed to the combined effects of biological, affective, and cognitive factors as well as sociocultural pressures of stereotypic conformity (Cyranowski et al., 2000; Hyde & Mezulis, 2020).

This sociocultural mechanism is compatible with both social rank and cognitive explanations of depressive symptoms and risks. For instance, the internalization of traditional gender roles might affect the social rank competition, encouraging boys, but not girls, to fulfill masculine roles with confidence and bravery (Bone et al., 2020) and elevate status concerns in peer groups (Cummins, 2005; Irons & Gilbert, 2005). The latter pressure prompts boys to be sensitive to social signals during adolescence (Irons & Gilbert, 2005), while girls of the same age period are also highly tuned in to interpersonal signals (Shih et al., 2006). The elevated vulnerability of girls to body‐image concerns and negative biases conducive to depressive symptoms are similarly related to traditional roles that suppress female agency (Cyranowski et al., 2000; Hyde et al., 2008). Therefore, increased pressure to internalize traditional gender roles should be linked to increased gender differences in depression susceptibility and the effect of corresponding protective factors. This theoretical extrapolation calls for empirical examination in future research.

Finally, societal pressure for gender‐role conformity is likely to be age‐specific: heightened pressure might be felt by adolescents who are 14–15 years old but not those who are 12–13 years old. It is important to note that the traditional gender roles are by no means invariant. Traditional gender stereotypes resulting from biological sex differences and sexual selection are attenuated in stable modern societies (Newson & Richerson, 2009; Zhu & Chang, 2019b, 2020), which should reduce the overall level of depression‐inducing stress for both genders. The present research provided a preliminary test of this idea through the employment of a representative sample with considerable regional diversity in social contexts. Specifically, we considered the local sex ratio and average years of education measured at the county level and their interlevel effects on the distributions of depressive symptoms between the genders. The results suggested that average years of education (but not sex ratio) were associated with lower levels of depressive symptoms (but mainly for the younger group), which is consistent with previous findings regarding the effects of education on depression (Ross & Mirowsky, 1989, 2006).

Despite its noteworthy findings, the present study has several limitations. First, the measures included in the current study are limited by the CFPS database and, therefore, do not cover all the potential social and cognitive protective factors against depression. As past reviews in the field (e.g., Cyranowski et al., 2000; Hyde & Mezulis, 2020) have pointed out, the gender difference in depression might stem from a complex interplay among mechanisms, such as sex differences in hormonal development (Naninck et al., 2011; Steiner et al., 2003), affective vulnerabilities (Hyde et al., 2008), genetic vulnerabilities (Eley et al., 2004; Uddin et al., 2010), and differential exposure or susceptibility to stress and negative events (Kendler et al., 2004; LeMoult et al., 2020; Shapero et al., 2013). These mechanisms are also moderated by sociocultural factors, such as media exposure and gender inequality (Hyde & Mezulis, 2020; Twenge et al., 2018). To better assess the true impacts of social and cognitive protective factors against depression during adolescence, future research should simultaneously examine other possible moderators of gender differences in depressive symptoms, such as early stressors, biological maturity, peer victimization, and negative events (Hilt & Nolen‐Hoeksema, 2014). Moreover, our research is limited by the small number of items related to peer popularity in CFPS. Due to its self‐reporting format, these items may not be the ideal mode of assessing peer popularity. Therefore, future research could benefit from the use of peer nomination or peer rating techniques to more accurately reflect adolescents' peer popularity.

Overall, our findings generate several practical suggestions regarding the intervention efforts to mitigate adolescent depressive symptoms. Our results support the benefit of encouraging positive peer interactions that might serve as a social protective network against depression. In cultural contexts (e.g., the Chinese culture) where parents sometimes play a predominant role in adolescents' social lives, granting adolescents more autonomy in seeking peer friendship and peer recognition might be especially helpful. In the meantime, for adolescents facing more acute tension between traditional and modernized gender roles (i.e., girls in rural areas with a stronger traditional cultural norm), boosting their self‐confidence and sense of control in independently solving real‐life problems should be especially beneficial. The gender imbalance in susceptibility to depression can be greatly attenuated if young females are encouraged to take control of their personal life and their environment.

## CONCLUSION

A comprehensive understanding of the gender difference in the susceptibility to depressive symptoms necessitates a simultaneous consideration of both social and cognitive factors. Our findings suggest that peer popularity functions as a protective factor against concurrent depressive symptoms for both adolescent boys and girls. It also had a stronger long‐lasting protective effect for girls than for boys 4 years later. By contrast, girls benefited more from the protective effect of self‐discipline against concurrent depressive symptoms than boys did during middle adolescence.

## FUNDING INFORMATION

This research has been funded by a General Research Fund of Hong Kong University Grants Committee (UGC) (Project 15608415) from the Research Grants Council (RGC) of Hong Kong SAR and a Chair Professor Grant (CPG2019‐00008‐FSS) from the University of Macau. The content is solely the responsibility of the authors and does not necessarily represent the official views of the funders.

## CONFLICT OF INTEREST STATEMENT

The authors declare that they have no conflict of interest.

## ETHICS STATEMENT

Ethics approval and patient consent are not applicable to this research.

## Supporting information


**DATA S1.** Supporting Information.

## Data Availability

The data that support the findings of this study are available on the Peking University Open Research Data website at https://opendata.pku.edu.cn/dataset.xhtml?persistentId=doi:10.18170/DVN/45LCSO.
