# Supplementary material for: Peer popularity and self‐discipline as protective factors against depressive symptoms in Chinese adolescents: Do boys and girls benefit equally?
Source: Psych J. 2023 Dec 17;13(1):66–78. doi: 10.1002/pchj.708 (PMC10917103; doi:10.1002/pchj.708)
Supplement: Supplementary file 1 — DATA S1. Supporting Information. [file PCHJ-13-66-s001.docx]

Supporting Information

This document contains additional analyses and detailed results for the study “Peer Popularity and Self-Discipline as Protective Factors against Depressive Symptoms in Chinese Adolescents: Do Boys and Girls Benefit Equally”.

This document includes: (1) a detailed description of candidate items and indices related to social and cognitive protective effects of depression and their correlations with the key variables (peer popularity and self-discipline) used in the final analysis, (2) a table (Table S1) comparing the demographic information (gender and provincial distribution) of the current subsamples and the original sample that it is drawn from, (3) a detailed description of the analysis of examining younger and older age groups using separate multilevel models, and two tables (Tables S2 and S3) containing results of these analyses.

**Candidate Items and Indices Related to Social and Cognitive Protective Effects**

As the CFPS study was not originally designed for the purpose of examining protective effects of peer popularity and self-discipline, researchers first combed through the child questionnaire in 2012 in search of relevant items. Only highly relevant items that cover most aspects of the construct that we intend to measure were used in our final analysis. Here, we report other candidate items and indices in CFPS child questionnaires that are related to social and cognitive protective effects and their correlations with the two key variables included in the final analysis.

Importantly, as a partial test of the construct validity of the two measures we used (peer popularity and self-discipline in 2012), we hypothesized that both peer popularity and self-discipline should be positively correlated with self-esteem (at least in 2012). In addition, peer popularity should be correlated with a higher number of online friends and stronger interpersonal relationships. Self-discipline, by contrast, should be correlated with higher personal control and a stronger belief that hard work will be rewarded in the future.

**Candidates for Both Protective Effects: Self-Esteem**

Individuals with low self-esteem generally face greater risks of depression (Orth et al., 2008, 2014). On the one hand, low self-esteem has been found to contribute to depressive symptoms via cascading effects of social motivation and social support (Masselink et al., 2018). One important source of self-esteem is the approval of peers (which boosts social rank and reduce status uncertainty; Gilbert, 2000). Recent research has shown that worries about rejections and loneliness are important mediators between low self-esteem and depression in Chinese adolescents (Zhou et al., 2020). On the other hand, low self-esteem might also be associated with negative cognitive biases that lead to maladaptive coping (such as rumination) and a lack of self-discipline (e.g., control, planning, and persistence), especially when the latter is highly predictive of academic achievement (Zimmerman & Kitsantas, 2014), a major source of self-esteem in competitive education system. Therefore, it is expected to be linked to both peer popularity and self-discipline. Participants’ self-esteem was measured in both 2012 and 2016 using an adapted Chinese version of Rosenberg Self-Esteem Scale (RSES; Rosenberg, 1965), containing 10 items (e.g., “I feel that I have a number of good qualities”, “I often think I am good for nothing [reverse-coded]”). The alpha coefficients for the 10 items were quite low, however (.54 in 2012 and .57 in 2016), probably due to the fact that the scale contained many reversely coded items. As expected, self-esteem in 2012 was positively correlated with both peer popularity (*r* = .25, *p* < .001) and self-discipline (*r* = .18, *p* < .001). However, self-esteem in 2016 was not correlated with either peer popularity (*r* = .08, *p* = .398) or self-discipline (*r* = .07, *p* = .424).

**Candidates for Social Protective Effects**

***Number of Friends on QQ in 2010.*** Participants’ number of online friends might reflect their popularity and social support (provided that they use social networking software). Therefore, a single question asking about participants’ number of friends on QQ (the leading social networking and messaging software in China in 2010) can be seen as an alternative measure of peer status. One shortcoming of this indicator of peer status is that only a minority of respondents (*n* = 306) responded to the question (probably due to the fact that social networking software had not been widespread in China in 2010). Although it correlated with peer popularity in the expected direction, this item was not significantly correlated with either peer popularity (*r* = .11, *p* = .058) or self-discipline (*r* = -.08, *p* = .186).

***Interpersonal Relationship in 2016*.** Participants’ self-perception of the overall quality of their interpersonal relationship was measured in 2016 using a single item “How strong are your interpersonal relationships”. Participants rated this item from 0 (*worst*) to 10 (*best*). It was moderately and positively correlated with peer popularity (*r* = .34, *p* < .001), but not with self-discipline (*r* = .12, *p* = .196).

**Candidates for Social Protective Effects**

***Personal Control***. Participants’ personal control was measured by four items in 2012: (1) I can't solve current problems (reverse-coded), (2) Sometimes I feel forced to do things to make a living (reverse-coded), (3) I'm in control of whatever happens to me, and (4) I feel helpless in my daily life (reverse-coded). The four items produced an alpha coefficient of .42, which makes them unsuitable for constructing a measure. Nevertheless, this index of personal control was positively correlated with self-discipline (*r* = .22, *p* < .001) as expected. It was also positively correlated with peer popularity with a lower magnitude (*r* = .20, *p* < .001).

***Reward for Hard Work***. A single item “Hard work will be rewarded in today's society” in the 2012 child questionnaire reflected participants’ belief in hard work. This item was positively correlated with self-discipline (*r* = .13, *p* < .001) as expected. It was also positively correlated with peer popularity with a lower magnitude (*r* = .10, *p* = .006).

Overall, most of our findings are in the expected direction, supporting the construct validity of the key measures. Compared with the measures used in the final analysis, however, all the other candidate items and indices suffer from various issues (e.g., low internal consistency, single item, lack of content validity).

**Separate Multilevel Models for Younger and Older Age Groups**

**Method**

***Participants***

Participants in the main analyses were divided into two subsamples: a younger subsample (12-13 years old) and an older subsample (14-15 years old). The younger and older subsamples consisted of 854 participants (419 females and 435 males) and 824 participants (418 females and 406 males), respectively.

***Measures***

Measures used in the follow-up analyses on younger and older age groups were the same as those used in the main analyses reported in the manuscript.

***Statistical Analyses***

At Level 1, we entered sex, health, peer popularity, self-discipline, and the interactions between sex and peer popularity, and between sex and self-discipline as individual-level predictors. At Level 2, we entered county-level sex ratio and average years of education, as well as the interlevel interaction between sex and sex ratio, and between sex and average years of education.

The same multilevel model was examined in the younger and older subsamples separately:

Level 1: *Depression* = β_0_ + β_1_*(sex) + β_2_*(health) + β_3_*(peer popularity) + β_4_*(self-discipline) + β_5_*(sex x peer popularity) + β_6_*(sex x self-discipline) + *r*Level 2: β_0_ = γ_00_ + γ_01_*(sex ratio) + γ_02_*(average years of education) + *u*_0_,

β_1_ = γ_10_ + γ_11_*(sex ratio) + γ_12_*(average years of education),

wherein βs and γs represent the intercepts and regression coefficients at the individual and county levels, respectively. *r* represents the individual-level residual and *u*_0_ represents county-level residuals, which indicate deviations from sample means.

**Results**

The results of the multilevel analyses for the younger and older subsamples are presented in Tables S2 and S3, respectively. The intra-class correlations (ICCs) for depression were .12 and .13 for the younger and older subsamples, respectively. Entering in the individual-level predictors accounted for 10% (from 0.0937 to 0.0842) and 12% (from 0.0855 to 0.0755) reductions in Level-1 residuals for depression compared with those of the baseline for the younger and older subsamples, respectively. Entering in the county-level predictors accounted for 19% (from 0.0131 to 0.0105) and 11% (from 0.0133 to 0.0118) reductions in Level-2 residuals compared with those of the baseline for the younger and older subsamples, respectively.

Like the multilevel analyses of depression based on the full sample, for both younger and older age groups, we found that individual-level health, peer popularity, and self-discipline were associated with lower depression. However, we only found significant sex difference (females reported higher depression than males) and interaction between sex and self-discipline (indicating that self-discipline had a stronger protective effect against depression for girls than for boys) in the older group (14-15 years old; Table S3), but not the younger group (12-13 years old; Table S2). In neither subsample did we find a significant interaction between sex and peer popularity. At Level 2, we found that for the younger group, but not the older group, average years of education was associated with lower depression, indicating that younger adolescents in better-educated counties were at lower risks for depression. At the same time, the interlevel interaction between sex and sex ratio had the tendency to be negatively associated with depression. This means that the sex difference (with boys scoring lower in depression than girls did) found in the older group was more drastic in counties with a more unbalanced sex ratio favoring boys.

References

Gilbert, P. (2000). The relationship of shame, social anxiety and depression: The role of the evaluation of social rank. *Clinical Psychology & Psychotherapy: An International Journal of Theory & Practice, 7*(3), 174–189. https://doi.org/10.1002/1099-0879(200007)7:3<174::AID-CPP236>3.0.CO;2-U

Masselink, M., Van Roekel, E., & Oldehinkel, A. J. (2018). Self-esteem in early adolescence as predictor of depressive symptoms in late adolescence and early adulthood: The mediating role of motivational and social factors. *Journal of Youth and Adolescence, 47*(5), 932–946. https://doi.org/10.1007/s10964-017-0727-z

Orth, U., Robins, R. W., & Roberts, B. W. (2008). Low self-esteem prospectively predicts depression in adolescence and young adulthood. *Journal of Personality and Social Psychology, 95*(3), 695–708. https://doi.org/10.1037/0022-3514.95.3.695

Orth, U., Robins, R. W., Widaman, K. F., & Conger, R. D. (2014). Is low self-esteem a risk factor for depression? Findings from a longitudinal study of Mexican-origin youth. *Developmental Psychology, 50*(2), 622–633. https://doi.org/10.1037/a0033817

Rosenberg, M. (1965). *Rosenberg Self-Esteem Scale (RSES)* [Database record]. APA PsycTests. https://doi.org/10.1037/t01038-000

Zhou, J., Li, X., Tian, L., & Huebner, E. S. (2020). Longitudinal association between low self‐esteem and depression in early adolescents: The role of rejection sensitivity and loneliness. *Psychology and Psychotherapy: Theory, Research and Practice, 93*(1), 54–71. https://doi.org/10.1111/papt.12207

Zimmerman, B. J., & Kitsantas, A. (2014). Comparing students’ self-discipline and self-regulation measures and their prediction of academic achievement. *Contemporary Educational Psychology, 39*(2), 145–155. https://doi.org/10.1016/j.cedpsych.2014.03.004

Table S1

*Demographics comparison of the subsample (12 to 15 years old adolescents in 2012) used in the current study and the original sample (child sample in 2012) it is drawn from.*

|  | Model 1 (percentage) | Model 2 (percentage) | Original Sample (percentage) |
| --- | --- | --- | --- |
| Gender |  |  |  |
| Female | 835 (49.8) | 632 (50.8) | 4,095 (47.5) |
| Male | 841 (50.2) | 611 (49.2) | 4,525 (52.5) |
| Province ID |  |  |  |
| 11 | 5 (0.3) | 3 (0.2) | 20 (0.2) |
| 12 | 9 (0.5) | 7 (0.6) | 53 (0.6) |
| 13 | 84 (5.0) | 63 (5.1) | 502 (5.8) |
| 14 | 62 (3.7) | 52 (4.2) | 309 (3.6) |
| 21 | 113 (6.7) | 90 (7.2) | 472 (5.5) |
| 22 | 13 (0.8) | 12 (1.0) | 93 (1.1) |
| 23 | 37 (2.2) | 29 (2.3) | 153 (1.8) |
| 31 | 47 (2.8) | 37 (3.0) | 294 (3.4) |
| 32 | 27 (1.6) | 19 (1.5) | 153 (1.8) |
| 33 | 23 (1.4) | 17 (1.4) | 93 (1.1) |
| 34 | 21 (1.3) | 15 (1.2) | 142 (1.6) |
| 35 | 15 (0.9) | 10 (0.8) | 121 (1.4) |
| 36 | 53 (3.2) | 39 (3.1) | 301 (3.5) |
| 37 | 70 (4.2) | 59 (4.7) | 287 (3.3) |
| 41 | 228 (13.6) | 191 (15.4) | 1,304 (15.1) |
| 42 | 18 (1.1) | 11 (0.9) | 99 (1.1) |
| 43 | 55 (3.3) | 37 (3.0) | 286 (3.3) |
| 44 | 206 (12.3) | 120 (9.7) | 1,041 (12.1) |
| 45 | 53 (3.2) | 41 (3.3) | 228 (2.6) |
| 50 | 18 (1.1) | 13 (1.0) | 93 (1.1) |
| 51 | 66 (3.9) | 57 (4.6) | 399 (4.6) |
| 52 | 117 (7.0) | 71 (5.7) | 465 (5.4) |
| 53 | 80 (4.8) | 53 (4.3) | 302 (3.5) |
| 61 | 27 (1.6) | 31 (2.5) | 183 (2.1) |
| 62 | 229 (13.7) | 166 (13.4) | 1,190 (13.8) |
| Total | 1,676 (100.0) | 1,243 (100.0) | 8,620 (100.0) |

Table S2

*Results of multilevel analysis for the younger age group: Full model with Time 1 depression as the dependent variable*

| Predictors | Coefficient | *SE* | 95% CI | | *t*-ratio |
| --- | --- | --- | --- | --- | --- |
|  |  |  | Lower limit | Upper limit |  |
| Level 1 (individual level) |  |  |  |  |  |
| Gender | 0.0091 | 0.0221 | -0.0341 | 0.0524 | 0.41 |
| Health | -0.0620 | 0.0122 | -0.0858 | -0.0381 | -5.09^***^ |
| Peer popularity | -0.0367 | 0.0098 | -0.0559 | -0.0176 | -3.76^***^ |
| Self-discipline | -0.1271 | 0.0339 | -0.1934 | -0.0607 | -3.75^***^ |
| Gender * Peer popularity | 0.0198 | 0.0135 | -0.0067 | 0.0463 | 1.52 |
| Gender * Self-discipline | 0.0365 | 0.0454 | -0.0525 | 0.1256 | 0.69 |
| Level 2 (county level) |  |  |  |  |  |
| Effect on intercept: Sex ratio | 0.0028 | 0.0018 | -0.0007 | 0.0063 | 1.15 |
| Effect on intercept: Average years of education | -0.0339 | 0.0125 | -0.0584 | -0.0094 | -2.71^**^ |
| Effect on gender slope: Sex ratio | -0.0008 | 0.0021 | -0.0050 | 0.0033 | -0.39 |
| Effect on gender slope: Average years of education | -0.0041 | 0.0150 | -0.0335 | 0.0253 | -0.27 |

^**^ *p* < .01 ^***^ *p* < .001

Table S3

*Results of multilevel analysis for the older age group: Full model with Time 1 depression as the dependent variable*

| Predictors | Coefficient | *SE* | 95% CI | | *t*-ratio |
| --- | --- | --- | --- | --- | --- |
|  |  |  | Lower limit | Upper limit |  |
| Level 1 (individual level) |  |  |  |  |  |
| Gender | -0.0842 | 0.0206 | -0.1245 | -0.0439 | -4.09^***^ |
| Health | -0.0344 | 0.0124 | -0.0586 | -0.0101 | -2.78^**^ |
| Peer popularity | -0.0307 | 0.0099 | -0.0501 | -0.0114 | -3.12^**^ |
| Self-discipline | -0.1165 | 0.0293 | -0.1739 | -0.0591 | -3.98^***^ |
| Gender * Peer popularity | -0.0103 | 0.0127 | -0.0351 | 0.0145 | -0.81 |
| Gender * Self-discipline | 0.0699 | 0.0330 | 0.0053 | 0.1346 | 2.12^*^ |
| Level 2 (county level) |  |  |  |  |  |
| Effect on intercept: Sex ratio | 0.0026 | 0.0022 | -0.0017 | 0.0068 | 1.18 |
| Effect on intercept: Average years of education | -0.0162 | 0.0149 | -0.0454 | 0.0130 | -1.09 |
| Effect on gender slope: Sex ratio | -0.0038 | 0.0020 | -0.0078 | 0.0002 | -1.84 |
| Effect on gender slope: Average years of education | -0.0122 | 0.0150 | -0.0415 | 0.0172 | -0.81 |

^*^ *p* < .05 ^**^ *p* < .01 ^***^ *p* < .001
